# Yeast as a cell factory: current state and perspectives

**DOI:** 10.1186/s12934-015-0281-x

**Published:** 2015-06-30

**Authors:** Martin Kavšček, Martin Stražar, Tomaž Curk, Klaus Natter, Uroš Petrovič

**Affiliations:** Institute of Molecular Biosciences, University of Graz, Humboldtstrasse 50/II, 8010 Graz, Austria; Faculty of Computer and Information Science, University of Ljubljana, Ljubljana, Slovenia; Department of Molecular and Biomedical Sciences, Jožef Stefan Institute, Jamova 39, 1000 Ljubljana, Slovenia

**Keywords:** Genome editing, Substrate utilization, Robustness development, Orthogonality, QTL

## Abstract

The yeast *Saccharomyces cerevisiae* is one of the oldest and most frequently used microorganisms in biotechnology with successful applications in the production of both bulk and fine chemicals. Yet, yeast researchers are faced with the challenge to further its transition from the old workhorse to a modern cell factory, fulfilling the requirements for next generation bioprocesses. Many of the principles and tools that are applied for this development originate from the field of synthetic biology and the engineered strains will indeed be synthetic organisms. We provide an overview of the most important aspects of this transition and highlight achievements in recent years as well as trends in which yeast currently lags behind. These aspects include: the enhancement of the substrate spectrum of yeast, with the focus on the efficient utilization of renewable feedstocks, the enhancement of the product spectrum through generation of independent circuits for the maintenance of redox balances and biosynthesis of common carbon building blocks, the requirement for accurate pathway control with improved genome editing and through orthogonal promoters, and improvement of the tolerance of yeast for specific stress conditions. The causative genetic elements for the required traits of the future yeast cell factories will be assembled into genetic modules for fast transfer between strains. These developments will benefit from progress in bio-computational methods, which allow for the integration of different kinds of data sets and algorithms, and from rapid advancement in genome editing, which will enable multiplexed targeted integration of whole heterologous pathways. The overall goal will be to provide a collection of modules and circuits that work independently and can be combined at will, depending on the individual conditions, and will result in an optimal synthetic host for a given production process.

## Background

The development of economically feasible and sustainable biotechnological processes as alternatives to oil based chemistry is one of the major goals of biobased economy. The success of this strategy will require efficient, robust and versatile cell factories but the improvement of currently used strains towards such platforms is hindered by the limitations of conventional methods for strain improvement. Synthetic biology is expected to provide means for engineering cell factories in a more efficient and controllable way.

Synthetic biology is regarded as the engineering discipline by which novel organisms can be constructed by assembling parts, devices and modules into systems. Proponents such as the iGEM (International Genetically Engineered Machine) foundation aim at a high level of standardization and foster the definition of a framework of rules for the conceptualization, design and manufacture of biological systems with predictable properties. A more practical, problem driven approach, however, sees synthetic biology as the field that makes use of advanced tools of genetic engineering, genomics and systems biology in order to programme and control a biological device and create new behaviour previously not found in that system. In contrast to the former approach, this description does not draw a clear line to other sciences; the designation of a strain as simply genetically engineered or synthetic seems rather arbitrary. Because of the complexity of biological systems, each problem is seen as unique and the occurrence of unpredictable effects of genetic modifications is expected, making attempts for standardization a futile effort. Irrespective of the ongoing debate about its definition [1, 2], there is general agreement that the success of synthetic biology in biotechnology will rely on the development of new methods to analyse and control cell systems and to allow for the targeted modification of genomes on a large scale. Hence, with the advance of recent genome editing techniques, of next generation sequencing and gene synthesis, and the merging of large datasets with modelling and novel bioinformatics tools, we expect that synthetic biology will significantly advance the design of industrially relevant strains for producing novel chemicals.

The yeast *Saccharomyces cerevisiae* is the most intensively studied unicellular eukaryote and one of the main industrial microorganisms used in the production of biochemicals. Apart from traditional applications in alcohol fermentations, baking processes and bio-ethanol production, *S. cerevisiae* is being used for the production of many industrially relevant biochemicals and for heterologous expression of proteins [3]. The potential of yeast as a powerful host for synthetic biology has already been successfully demonstrated by both basic research, namely the de novo synthesis of a complete chromosome [4], and the application-oriented engineering of complex pathways, like the synthesis of amorphadiene and vanillin [5, 6].

There are two basic strategies for developing a production host for a biotechnological process. In the first, a suitable host can be selected from a large number of species based on its performance regarding parameters such as product yield, productivity, and tolerance to the product or other environmental stressors (e.g. pH, temperature, salt). In many cases, targeted optimization of such a host is not possible because the tools for genetic analysis and engineering in that species are not available, leaving only evolutionary optimization or random mutagenesis to produce optimized strains. The second possible strategy is to start with a well-known species such as *S. cerevisiae* and optimize it for the desired product and required bioprocess conditions. Many examples for this strategy exist, but species-specific traits often hinder the development of hosts with high productivity and yields close to theoretical limits. Nevertheless, *S. cerevisiae* is the host of choice in many cases, due to the vast array of tools for genetic engineering and to the immense range of knowledge about all aspects of yeast biology. In this review, we will highlight recent achievements in engineering *S. cerevisiae* for biotechnological processes, with focus on advanced synthetic biology tools for genome editing, pathway control and for the analysis and transfer of industrially relevant traits. Moreover, we will discuss further developments that will be required to establish *S. cerevisiae* as one of the chassis in synthetic biology and as a platform for future cell factories. Finally, we will present an approach whereby both aforementioned strategies for strain development can be combined in a synergistic manner, to obtain a platform strain that can be individually adapted according to the requirements of a specific process.

## Development of genome editing tools

Traditional DNA editing techniques, such as transformation and deletion of genes by homologous recombination, have been readily feasible for many years in *S. cerevisiae.* The use of Cre recombinase or other recombination based approaches, like the 50:50 method [7], allow for marker recycling and performing *delitti perfetti*, leaving no foreign DNA in the yeast genome. Although such techniques are currently an important tool for synthetic biology, they are relatively time consuming and therefore not suitable for the introduction of whole heterologous metabolic pathways or deletions of several genes in a reasonably short time. In the last few years, new approaches like Zinc finger nucleases [8], Yeast Oligo-Mediated Genome Engineering (YOGE) [9], transcription activator like (TAL) effector nucleases [10] and the CRISPR-Cas system (clustered regularly interspaced short palindromic repeats) [11, 12] have been developed for deleting or inserting genes and for controlling gene expression. The main advantages of these tools over traditional techniques lie in their efficiency, accuracy and speed. They all rely on site-specific endonucleases forming a double strand break that is repaired either with non-homologous end joining or homology-directed repair. The versatility of the methods stems from the ability to customize the DNA binding domains, allowing for site-specific genome editing. From the techniques mentioned, the use of CRISPR, together with the site specific Cas9 endonuclease, appears as the most promising tool for editing a genome at any number of different loci in a short time. Remarkably, DiCarlo et al. reported a close to 100% recombination efficiency of a linear dsDNA after transformation, together with a plasmid bearing the guiding RNA [11]. Similar approaches were already used for multiplex deletions of up to five genes [13, 14]. Hence, the CRISPR/Cas9 tool makes the use of marker-flanked integration cassettes obsolete and paves the way for rapid and efficient genome editing.

Other approaches make use of the high recombination efficiency of *S. cerevisiae* by simultaneous transformation of a recipient strain with several different integration cassettes. One successful strategy used up to four cassettes, with each one containing two genes under the control of the bidirectional GAL1/10 promoter and a phleomycin resistance marker, and with targeted chromosomal δ sites of transposons. An alternative approach, the DNA assembler technique, is based on the in vivo recombination of overlapping DNA sequences. All genes of a pathway together with a marker were amplified by PCR with extension primers that resulted in overlapping sequences at the 3′-end of one gene and the 5′-end of the next one. The 5′-end of the first and the 3′-end of the last cassette bore sequences homologous to sequences in the chromosomal δ sites. For both strategies, pathways with up to eight genes were successfully assembled in one round of transformation [15, 16]. These approaches reduce the risk of undesirable background mutations in the recipient strain, since only one transformation is required. The use of δ sites, which are highly abundant in the genome, probably increases the efficiency of these methods. On the other hand, the exact positions of the integrated cassettes are not known and their numbers could vary, either as a result of different integration efficiencies or, later on, due to duplication of transposons. However, these drawbacks may be overcome when such methods are combined with one of the abovementioned techniques of targeted integration through endonucleases at unique loci. Indeed, a recent study demonstrated the potential of such a combination with the successful co-transformation of 15 linear DNA fragments, their in vivo assembly into 3 genes and targeted integration by Cas9 and gRNA [17]. The rate of positive transformants was too low to be used for a marker-free strategy. Nevertheless, if such approaches prove to be generally applicable, extensive genetic engineering will no longer be limited by the time consuming introduction or deletion of one gene after the other. Furthermore, it will be possible to obtain strains with different combinations of genetic modifications by single transformations, e.g. by co-transformation of alternative heterologous genes coding for the same enzymatic function. The resulting strains can then be tested for performance regarding a desired trait. The drawback of the CRISPR/Cas9 technique, especially in the context of multiplex engineering, is the requirement for a specific gRNA plasmid for each target locus (or for one plasmid with several gRNA sequences). However, we expect that gRNA libraries will soon become available. Such collections could provide plasmids with guiding sequences that target loci with high transcriptional activity on different chromosomes and could be used for the simultaneous insertion of several genes of a heterologous pathway.

The currently most ambitious project in yeast synthetic biology is the complete de novo synthesis of all 16 chromosomes, Sc2.0. In this effort, all nonessential genes will be flanked by *loxP* sites, allowing for random deletion of genes upon expression of Cre recombinase and on screening for viable strains with improved characteristics for a selectable trait. Furthermore, one of the three stop codons will be eliminated from the genome in this project. In the future, an orthogonal codon could be used for the targeted incorporation of an alternative amino acid, thereby altering protein properties. Such a recoded genome will also enable the development of efficient biocontainment strategies as the free codon can be used to engineer orthogonal auxotrophies in cell factories to minimize risk in the case of accidental release and allow for processes to be carried out in open bioreactors [18, 19]. Although this project is at its very beginning, with one synthetic chromosome completed [4], the consortium plans to finish all additional chromosomes until 2019 [20]. It is thus not yet clear whether replacement of all chromosomes with their synthetic analogues will be possible, but Sc2.0 will certainly provide new knowledge about the genetics of yeast and genome editing. The rearrangement of tRNAs and elimination of transposable elements might be seen as one of the most interesting aspects of this project. The proponents argue that this strategy will lead to genome stabilization [4]. However, increased instability of the “party chromosome”, an additional chromosome that will bear all tRNA coding sequences, has to be expected. If these alterations will indeed result in overall increased genome stability, such a strategy could become useful to improve the robustness of cell factories.

## Development of orthogonal systems

**Figure 1:**
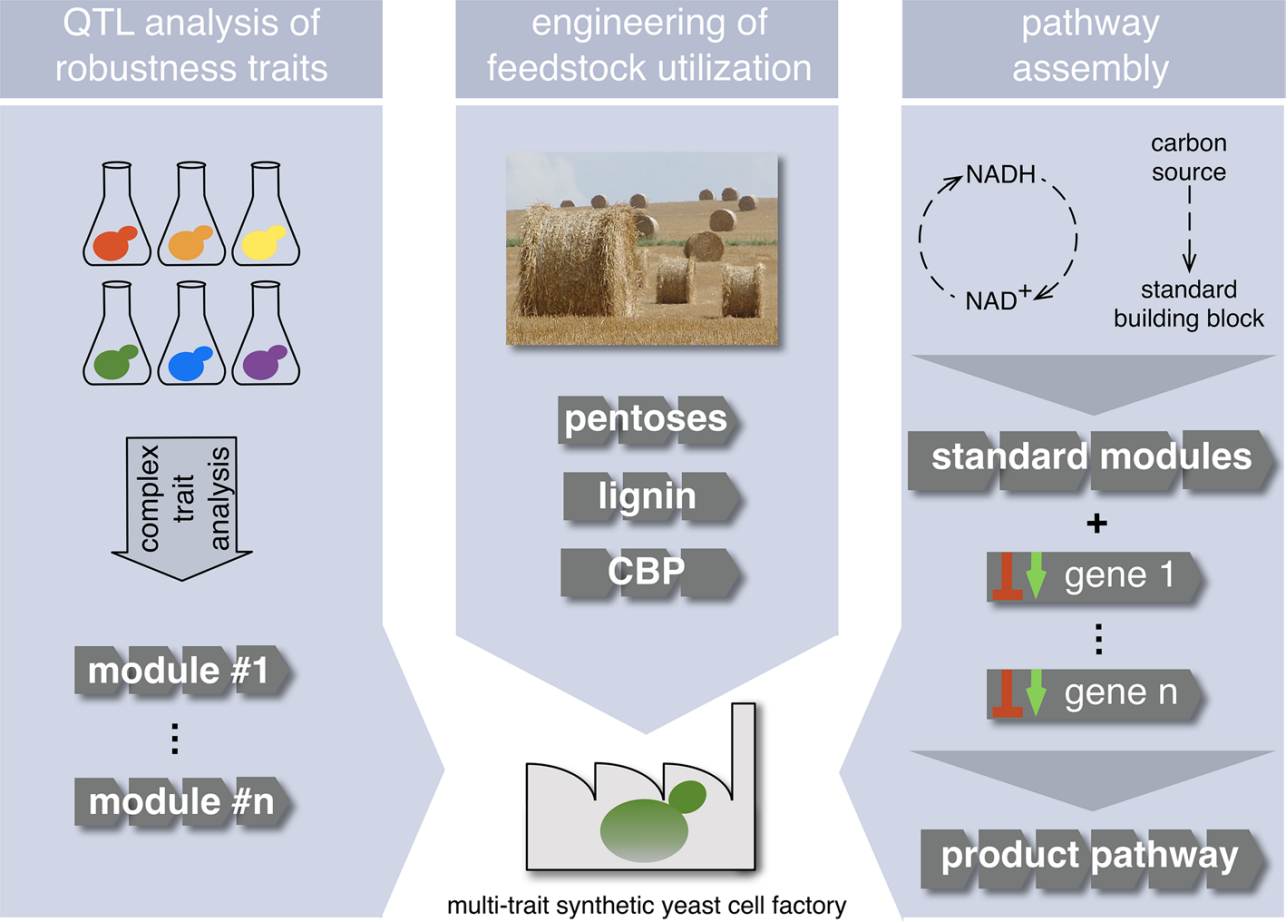
The assembly of a multi-trait yeast cell factory. The future yeast cell factory strains will require combinations of several traits, each of which will be encoded by a specific genetic module (depicted by strings of *arrows*) engineered using the state-of-the-art synthetic biology approaches, such as marker-free multiplex genome editing and orthogonal promoter libraries. In the future, individual “ready-to-use” modules should become available for fast transfer of the desired traits to the recipient strain in any combination. The order in which the respective modules are introduced will depend upon the specific conditions/requirements. *Left* Depending on the biotechnological process, different robustness traits will have to be introduced into the starting strain (e.g. tolerance to extreme pH, osmotic stress, organic acids or other toxic substances). Following the isolation of strains with superior performance with regard to specific traits (depicted by *different colours*), causal genes can be identified by polygenic trait analysis and/or by bioinformatics methods, as described in the text. *Centre* Optimization of the strains for efficient utilization of renewable feedstocks is another important aspect in engineering multi-trait yeast cell factories. Utilization of pentoses, especially xylose, and in the future also of lignin, will enable more cost-effective production of biochemicals. The next generation of yeast cell factories will be capable of consolidated bioprocessing (CBP), as described in the text. *Right* The biosynthesis pathway for the desired product could consist of a number of endogenous and/or heterologous genes. These genes will be combined with standard modules that will provide common building blocks or contribute to cofactor balance (The image in “B” is a detail from “Champs DSC01354" by Daplaza, licensed under CC BY-SA 2.5).

One of the central aims of synthetic biology is to apply classical engineering principles to the development of strains. This includes the concept of orthogonality that requires a biological system to be divisible into modules that are independent from each other and can therefore be engineered individually, without consideration of other modules and with predictable outcome (Figure 1). In contrast, system-wide approaches like systems biology and the various -omics techniques teach us that virtually each part of a biological system could be responding to changes in another part, with spatial, temporal or functional causalities that are often difficult or impossible to predict with our current knowledge. Hence, absolute orthogonality may, in the near future at least, not be achievable for biological devices.

Approaches in this field that go beyond theoretical considerations include the synthetic yeast strain with an orthogonal codon on the DNA level (see above), the engineering of aminoacyl-tRNA synthetases [21], riboregulators [22], and orthogonal ribosomes [23] on the translational level, and enzymes with specificity for orthogonal co-factors like xanthosine 5′-triphosphate [24] on the level of enzyme activity. Such studies will undoubtedly contribute considerably to the implementation of orthogonality in synthetic biological systems. For biotechnology applications, however, it seems unlikely that any of these approaches will be of relevance, even in the medium term, because they are too far from translation into production hosts and because there is no obvious, industrially relevant, advantage of such systems over existing, non-orthogonal, hosts. In contrast, orthogonality on the level of pathway control would be very important for the optimization of strains. Transcriptional control is the most common means of regulating the flux through a pathway. In *S. cerevisiae*, the majority of promoters that are used for control of expression are endogenous promoters that respond to the concentration and type of the carbon source [25]. Besides, inducible systems have been developed that respond to amino acid availability (*MET15*), to metal ions (*CUP1*) or to antibiotics, like the well-established *tetR/tetO* system [26], but, similar to the carbon source dependent promoters, these modules cannot be used for orthogonal expression. Regarding the possible complexity of the network that has to be controlled in a synthetic organism, it would be most advantageous to have a set of transcription factors (TFs) that are identical in their DNA binding properties but activatable by different chemical inducers, possibly at extremely low concentrations and in a titratable manner. Because of the requirement for orthogonality, these inducers are expected to cause no other response in yeast. Promising examples for transcriptional orthogonality are the estradiol-inducible chimeric TF [27], the retinoid X receptor [28], and the bacterial quorum sensing TF luxR [29], which has not yet been tested in *S. cerevisiae*. The advantage of these systems is that the ligand binding domain can be mutated to bind, with high specificity, a number of different, although structurally related, ligands. Starting from one TF, such mutations would result in a series of variants with identical binding but different activation domains. Most such approaches in yeast, however, use the binding domain of the *GAL4* TF. For this reason, although these engineered systems work independently of galactose induction, they interfere with genes of this regulon and activate their transcription upon induction. To obtain modules that can be controlled orthogonally, foreign binding mechanisms will have to be used. As noted in the previous section, genome editing tools can be used, not only for targeted modifications of DNA, but also for the control of gene expression. For example, the TAL effector can be designed to bind to any specific DNA sequence with high specificity; it has already been demonstrated to work as a repressor of a constitutive promoter in yeast [30]. A CRISPR/Cas9 system with a mutated and inactive endonuclease can be used in a similar manner [31]. If the DNA binding domains of these bacterial systems can be combined with chemically gradable activation domains, they will probably provide a highly efficient tool for the fine-tuned orthogonal expression of synthetic pathways.

## Predicting improved robustness and stress tolerance

Biotechnological processes often require strains that are tolerant to one or several stress conditions from a broad spectrum, like extreme pH, high temperature, osmotic pressure, shearing forces, organic acids and toxic substances. Most of these properties are complex traits, encoded by several genes (Figure 1, left). Basic genetic analysis methods therefore fail to characterize the underlying genetic network, and efforts to optimize one of these traits traditionally rely on adaptive evolution or breeding strategies. The possibilities of whole genome sequencing at low cost and in a comparably short time have now opened the way for the use of advanced genome analysis tools like quantitative trait loci (QTL) analysis to identify, at least under some conditions, all causative genes for a certain trait [32] or even several different genetic combinations giving rise to the same phenotype [33]. Extreme QTL (X-QTL) analysis and intercross QTL (iQTL), which have recently been improved greatly, provide sensitivity and detection of even modest changes in a trait to a single gene or even nucleotide level, simultaneously covering all causal loci contributing to heritability of the trait [32, 34].

These complex genetics techniques use the advantages of next generation sequencing capabilities to greatly increase the number of segregants that can be tested, but also produce more data that cannot be interpreted with traditional computational tools. To infer causative relationships between loci/genes and complex traits, novel data integration models are applied. While it is a common practice to apply conventional statistical testing on SNP polymorphisms for QTL discovery, it has been observed that a larger number of additional *known* factors can influence the resulting phenotype [35–37]. Existing methods can be tailored to integrate additional known factors, such as: the expression levels of transcripts, proteins or metabolites, transcription factor binding data, gene annotations and metabolic pathways, environmental conditions, experimental procedures as well as related phenotypes themselves. Complementary to observable factors, probabilistic Bayesian frameworks can be applied in order to account for unobservable, *hidden* factors [38], e.g. cell culture conditions, uncovering additional QTL-related features and further improving detection. A collection of data sources thus constitutes a comprehensive genome-wide polygenic trait detection model, useful in both laboratory and industrial experimental design. Current machine learning research is focusing on efficient data integration algorithms, such as matrix factorization [39], Bayesian networks [40] and multivariate regression [41], exploiting multiple data sources and accounting for both known and hidden factors. The additional information gained from multiple views on the data will improve various learning tasks, including gene prioritization, prediction or classification, potentially reducing the number of experimental assays required to investigate a given hypothesis. Another related and important property of these methods is the ability to include a variety of data sources without explicit tailoring of methods for each data type. This could prove particularly important, since synthetic biology is constantly providing new means for manipulating and designing industrially relevant strains, such as pathway engineering, RNA parts and devices, protein engineering or design of novel gene regulatory networks [42], with the potential of generating large quantities of experimental data of a strain response to single or multiple manipulations.

The aforementioned methods enable integration of data on mutations, environmental conditions [43, 44] and strain efficiency [45, 46]. As such, they will aid in the discovery of promising combinations of genetic manipulations, strains and environmental conditions to achieve multiple engineering objectives such as yield or breeding efficiency [44], as well as meeting productivity, efficiency or robustness constraints. Combining the knowledge of causative gene networks and metabolic models, it is possible to predict side effects and other trade-offs associated with manipulations. The main consequence of applying integrative mathematical modelling is, and will remain, the significant speed-up gained by informed manipulations comparing over the traditional trial-and-error approach.

## Improvement of the substrate spectrum

The metabolism of *S. cerevisiae* is specialized for the utilization of glucose, fructose and its disaccharide sucrose. In the emerging era of bioeconomy, however, microbial cell factories will have to efficiently utilize more sustainable, cheaper and generally available carbon sources, especially lignocellulose [47, 48] (Figure 1, centre). *S. cerevisiae* cannot directly utilize cellulose and therefore pretreatment is required to release glucose (see below).The second most abundant monosaccharide in plant biomass is xylose, but the rate of xylose metabolism in currently used laboratory and industrial yeast strains is too slow to be of use in a biotechnological process, especially because of too low xylitol dehydrogenase (XDH) activity [49, 50]. Adaptive evolution experiments resulted in strains with increased XDH activity and significantly shorter doubling times on xylose as the sole carbon source [51]. Moreover, several wine strains have been found that harbour in their genomes a previously unknown XDH-encoding gene named *XDH1* [52], indicating that it may be possible in the future to engineer an efficient endogenous xylose utilization pathway. Still, currently the most efficient utilization of xylose as the carbon source requires introduction of heterologous pathways, most often bacterial xylose isomerase. To construct the currently most efficient pentose fermenting strain published, a cassette of 13 genes, coding for enzymes of the xylose and arabinose utilization pathways and of the pentose phosphate pathway, was inserted into the genome of an industrial strain. Together with mutagenesis, genome shuffling and evolutionary engineering, the authors obtained a strain that produced 32% more ethanol from lignocellulosic hydrolysates than the parent strain. Although at lower consumption rates than for glucose, this synthetic strain fermented xylose to ethanol with yields close to the theoretical maximum [53].

The knowledge from decades of research on the utilization of lignocellulose-derived sugars is now being transferred to yeast-based processes and several second generation ethanol plants have recently started production or will do so in the near future [54]. The cell factories in these plants, however, mainly utilize glucose and xylose, whereas most other components of lignocellulose are regarded as waste. This is especially true for lignin, which composes up to 40% of lignocellulosic biomass. Although many of the enzymes required for lignin degradation are known, only little work is being done to transfer this knowledge to yeast and to develop lignin utilizing strains. Given the heterogeneity and complexity of lignin, the efficiency of its degradation pathways in *S. cerevisiae* is currently not predictable. Nevertheless, lignin utilization should be seen as a long-term goal for the sustainable utilization of renewable feedstocks.

Since direct utilization of lignocellulosic material as a feedstock for yeast is not yet possible, thermal, chemical and/or enzymatic pretreatments are required to separate the polymers that constitute lignocellulose and release the sugar monomers. Subsequent detoxification is often necessary to remove pretreatment derived inhibitory substances—especially acetic acid, formic acid, furan derivatives and phenolic compounds. Mechanisms conferring tolerance to such inhibitory substances can be predicted and engineered in yeast (see below), but development in this field has until now brought only limited success, although with promising predictions for the future [55]. Therefore, despite the lower costs of the raw materials, the second generation biofuels are currently still more expensive than the bioethanol produced from corn or sugar cane. To make cellulosic ethanol price-competitive, and to pave the way for the use of lignocellulose as raw material also for other biotechnological processes, novel solutions will be required. The most promising ones aim at so-called third generation processes, enabled by consolidated bioprocessing (CBP). CBP requires a single organism capable of biomass hydrolysis and bio-product production [56]. In terms of scientific approaches, development of such strains calls for merging of the fields of heterologous expression of cellulases and xylose fermentation. In addition, strains for CBP will be needed that are capable of maintaining the correct levels of expression of heterologous proteins—often for both cellulose hydrolysis and specific product biosynthesis—and that are resistant to the high temperatures required for most efficient biomass hydrolysis, and to the inhibitory compounds generated in this process. Hence, the assembly of strains for CBP is one of the most ambitious goals in the development of cell factories because it will require integration of knowledge from all the fields referred to above.

## Enhancement of the product spectrum

The specialization of *S. cerevisiae* on fast fermentation of sugars is the basis for its use in the production of alcoholic beverages and biofuel and in the baking industry. At the same time, aerobic ethanol fermentation (also called the Crabtree effect) is one of the main obstacles to obtaining high yields in processes aimed at producing bulk products other than ethanol. Indeed, sustainable and cost-effective production of many commercially important metabolites cannot be achieved in *S. cerevisiae* as long as most of the carbon source is converted to ethanol. Hence, a stable conversion of *S. cerevisiae* physiology to respiration in the presence of high sugar levels, allowing efficient use of the substrate, is an important prerequisite for its use in high yield production processes.

Most attempts to eliminate the Crabtree effect in *S. cerevisiae* focus mainly on a reduction of the normally high glycolytic flux, because it is assumed that the degree of fermentative activity is a function of the rate of glucose catabolism [57, 58]. A promising approach towards this aim is deletion of the seven major hexose transporters and their replacement with a chimeric transporter. These modifications result in reduced growth rates, but increased biomass yields and absence of ethanol production at moderate glucose concentrations [58]. Whether this strain is sufficiently robust to be used in biotechnological processes remains to be shown but its superior properties in heterologous protein production have already been demonstrated [59]. On the other hand, aerobic fermentation and concurrent ethanol excretion can be seen as a trade-off for conditions that are less prone to contamination and require less aeration than processes with respiratory organisms. Furthermore, if the post-diauxic ethanol consumption phase can be integrated into the production process, the product yield will be improved considerably. Importantly, the production of ethanol can be replaced with other pathways that balance the reducing equivalents originating from glycolysis. This has been successfully demonstrated with strains producing 2,3-butanediol [60] or lactate [61]. Hence, if a production pathway includes a reductive NADH-dependent step, it might be possible to eliminate ethanol excretion.

Synthetic biology approaches have resulted in recombinant yeast strains for the production of metabolites that are not normally produced by this species (Figure 1, right). Prominent examples are amorphadiene [6] and vanillin [5]. Other studies aiming at the production of polyketides [62], isoprenoids [63, 64], steviol components [65] and opiates [66] suggest that there are no obvious limits to the type of biochemicals that can be produced in yeast. Many of the above mentioned products are derived from reductive pathways, mostly dependent on NADPH. Therefore, synthetic biology approaches aiming at high yields will have to address not only the synthesis pathway itself, but also the system-wide redox balance of the host. Computational modelling approaches will likely become an important tool in the future to predict optimal engineering strategies for redox cofactor balance at high rates of product synthesis. The yeast community provides a continuously updated network reconstruction of yeast metabolism [67], which allows for metabolic modelling with simple flux balance analysis or with more complex methods like elementary flux mode analysis [68] and minimal metabolic behaviours [69]. Future efforts in this area will have to be focused on the integration of experimental data, like quantitative transcriptome, proteome and metabolome data, in the models to improve predictions, since consideration of circumstantial data can both improve analysis and provide new leverage for in vivo and in silico design. This will require high quality data sets, which are still rather rare. Furthermore, the endogenous basic network reconstruction will have to be extended by a meta-genomic library of exogenous reactions to enable optimal pathways for maximum productivity and cofactor-balanced metabolism to be predicted.

Orthogonality, although theoretically worthwhile, is difficult to achieve in biological systems. Nevertheless, it should be seen as one of the goals of synthetic biology in order to design modules for the supply of intermediate metabolites and cofactors with general applicability in metabolic engineering that are independent of the overall pathway which they serve. Examples would be circuits for regenerating redox cofactors or efficient modules for providing central building blocks such as 2-carbon (acetyl-CoA), four carbon (oxaloacetate), and six carbon (citrate) metabolites that can be of use in engineering many different pathways. Such readily available circuits would considerably facilitate metabolic engineering and enable faster expansion of the product spectrum of yeast.

## Perspective: combining polygenic trait analysis with synthetic biology

Several genetic modules [70–75] show that mutations in genes encoding regulatory proteins enable expression of the studied trait. Using synthetic biology tools to engineer trait-specific genetic modules can thus be seen as a step towards synthetic regulatory circuits with the ability to drastically increase the productivity of yeast strains. Since synthetic biology is not yet at the level of de novo organism design, this is, in our opinion, one of the most promising approaches towards a platform for yeast-based cell factories with an unprecedented lack of constraints, that are applicable to a wide range of biosynthetic pathways. Productive combination of genetic modules for tolerance to several stress factors will likewise remove some of the current bottlenecks in the development of new and more sophisticated cell factory-based processes. Although it remains to be seen how and if genetic interactions will occur between introduced causal genes, and how they would affect the expression of a combination of traits, we expect that the resulting complex cell factories will play a crucial role in the biotechnology of tomorrow, providing flexibility and robustness for novel processes.

Improved substrate spectrum, enhanced product spectrum and increased stress tolerance and robustness are the main demands for the future cell factories that will be used in biorefineries (Figure 1). As described above, these traits are almost exclusively polygenic. Recently developed polygenic trait analysis methods, such as X-QTL and iQTL, enable identification of complete sets of causal alleles, i.e. genetic modules, for the desired traits. These traits are present in natural strains, and yeast biodiversity is therefore an attractive genetic pool for bioeconomy. The development of synthetic biology toolboxes, on the other hand, enables genetic modules to be inserted into platform strains. We foresee an approach in which the latest developments in complex genetics are combined with expertise in synthetic biology, with the aim of combining several genetic modules in single strains (Figure 1). This new approach will make it possible to combine multiple beneficial traits within a single organism, which is not possible in the current state-of-the-art. Specific combinations of traits could result in strains custom-made for requirements of specific processes. Such cell factories should have a big potential for future biorefineries where several sources of feedstock and several different products will be used/produced within a relatively short time intervals. It is the ability to transform different molecules into pre-defined end products which makes the multi-trait cell factories important within the value chain concept of bioeconomy. In addition, as multi-trait cell factories will contain genetic modules comprising heterologous genes, the gap between biotechnological exploitation of *S. cerevisiae* and so-called non-conventional species will be diminished, since we can envision that some cell factories could make use of *S. cerevisiae* only as a chassis, whereas the specific biotechnologically relevant traits will come from a number of different organisms.

## Conclusions

New technologies for the analysis of whole genomes and for large scale DNA editing have the potential to revolutionize biotechnology. The engineering of production strains will no longer be restricted by the length or complexity of a pathway and the use of computational and -omics tools will enable more accurate prediction and prevention of undesirable side effects in the design phase. Due to its current importance in biotechnology and its immense knowledge base, *S. cerevisiae* will most probably also play a role as a chassis for synthetic biology and for the next generation of production hosts in biotechnology. The well-developed toolbox for the analysis of yeast, both on the single gene level and in -omics and systems biology techniques, is an important advantage of this organism. Combination of the recent developments in the fields of synthetic biology with polygenic trait analysis provides a means to engineer traits for increased stress tolerance and robustness, improved substrate spectrum, and enhanced product spectrum. However, synthetic biology research in yeast lags behind that in other organisms, especially *E. coli*. Indeed, there are only few efforts to standardize engineering and to improve the collection of biological parts for yeast are only limited. To establish *S. cerevisiae* as a promising candidate as a chassis in synthetic biology and future biotechnology, research will have to catch up in several areas, such as the development of orthogonal parts for expression control and genome editing. These are not only aspects of academic relevance but also prerequisites for the rapid and predictable engineering of readily controllable synthetic hosts.
